# Differential Regulation of Amyloid Precursor Protein/Presenilin 1 Interaction during Ab40/42 Production Detected Using Fusion Constructs

**DOI:** 10.1371/journal.pone.0048551

**Published:** 2012-11-12

**Authors:** Naoyuki Sato, Masayasu Okochi, Mitsuru Shinohara, Gopal Thinakaran, Shuko Takeda, Akio Fukumori, Motoko Shinohara-Noma, Mari Mori-Ueda, Hizuki Hamada, Masatoshi Takeda, Hiromi Rakugi, Ryuichi Morishita

**Affiliations:** 1 Department of Clinical Gene Therapy, Graduate School of Medicine, Osaka University, Yamada-oka, Suita, Japan; 2 Department of Geriatric Medicine, Graduate School of Medicine, Osaka University, Yamada-oka, Suita, Japan; 3 Department of Psychiatry and Behavioral Proteomics, Graduate School of Medicine, Osaka University, Yamada-oka, Suita, Japan; 4 Department of Neurobiology, University of Chicago, Chicago, Illinois, United States of America; “Mario Negri” Institute for Pharmacological Research, Italy

## Abstract

Beta amyloid peptides (Aβ) play a key role in the pathogenesis of Alzheimer disease (AD). Presenilins (PS) function as the catalytic subunits of γ-secretase, the enzyme that releases Aβ from ectodomain cleaved amyloid precursor protein (APP) by intramembrane proteolysis. Familial Alzheimer disease (FAD)-linked *PSEN* mutations alter APP processing in a manner that increases the relative abundance of longer Aβ42 peptides to that of Aβ40 peptides. The mechanisms by which Aβ40 and Aβ42 peptides are produced in a ratio of ten to one by wild type presenilin (PS) and by which Aβ42 is overproduced by FAD-linked PS variants are not completely understood. We generated chimeras of the amyloid precursor protein C-terminal fragment (C99) and PS to address this issue. We found a chimeric protein where C99 is fused to the PS1 N-terminus undergoes *in cis* processing to produce Aβ and that a fusion protein harboring FAD-linked PS1 mutations overproduced Aβ42. To change the molecular interactions within the C99-PS1 fusion protein, we made sequential deletions of the junction between C99 and PS1. We found differential effects of deletion in C99-PS1 on Aβ40 and 42 production. Deletion of the junction between APP CTF and PS1 in the fusion protein decreased Aβ40, while it did not decrease Aβ42 production in the presence or absence of FAD-linked PS1 mutation. These results are consistent with the idea that the APP/PS interaction is differentially regulated during Aβ40 and 42 production.

## Introduction

Alzheimer disease (AD) is a neurodegenerative disease characterized by the presence of senile plaques, of which beta amyloid peptide (Aβ) is the primary component [1]. Aβ is considered to be involved in the pathogenesis of AD, because familial AD (FAD) has been linked to mutations in the genes that encode amyloid precursor protein (APP) [2], [3], [4] and presenilin (PS) [5], [6], [7]. Mutations in these genes result in most cases in a relative increase in the production of Aβ42 [8], [9], which is the predominant form found in senile plaques [10]. Genetic ablation revealed that PS is required for cleavage of the APP C-terminal fragment (CTF), which is generated by α- or β-secretase cleavage of full-length APP within the extracellular/luminal domain [11], [12]. It is clear now that PS proteins are the catalytic subunits of γ-secretase [13], [14], [15]. Identification of the mechanisms by which intramembrane cleavage is performed has been a topic of intensive investigation. Two aspartate residues in the predicted transmembrane domains 6 and 7 in PS are essential for γ-secretase activity [13]. Moreover, at least three molecules, termed anterior pharynx defective phenotype 1 (APH-1), PS enhancer 2 (PEN-2), and nicastrin (NCT), are required for γ-secretase activity [14]. The electron microscopic structure of purified, active γ-secretase complex revealed a pore in the structure [16], [17], but the structural resolution was too low to allow predictions about the working mechanisms. Indeed, it is unknown how Aβ40/Aβ42 are produced in a ratio of ten to one by wild type PS and how Aβ42 is overproduced by FAD-linked PS variants. The nature of APP-CTF/PS interaction during Aβ40 and 42 production is not fully understood.

In the present study, we examined the mechanism by which Aβ40 and Aβ42 are produced. To address this issue, we generated a series of constructs encoding chimeric proteins where APP CTF is fused to the N-terminus of PS, taking advantage of previous findings that deletion of the N-terminal domain of PS does not affect γ-secretase activity [18]. Here, we show that fusion of APP CTF to the N-terminus of PS produces Aβ and that fusion of APP CTF to FAD-linked presenilin 1 variants overproduces Aβ42(43). Deletion analysis of the junction between APP CTF and PS1 in the fusion protein suggests differential regulation of APP/PS interaction underlying Aβ40 and 42(43) production.

## Methods

### Constructs

To generate an expression plasmid encoding the C99-PS1 fusion protein, expression constructs were engineered by PCR-based methods. Briefly, the first PCR-1 was performed with C99 cDNA as a template and the primers, oligonucleotide C99-PS1-1f (5′-ACCCAAGCTTCACAGCTAGCGCACTCGGTG-3′), which is complementary to a sequence encoding upstream of the signal sequence at the N-terminus of C99 and includes a NheI site, and oligonucleotide C99-PS1-1r (5′-GTGGTTGTCCTCAGACTGCTCAAAGAACTTG-3′), which encodes the C-terminus of C99 without the last three amino acids of C99 and stop codon and the N-terminus of PS1 without the first 16 amino acids. The first PCR-2 was performed with PS1 cDNA as a template and the primers, oligonucleotide C99-PS1-2f (5′-AAGTTCTTTGAGCAGTCTGAGGACAACCAC-3′), which is complementary to oligonucleotide C99-PS1-1r, and oligonucleotide C99-PS1-2r (5′-GGACCTTTCCAGTGAATGGAAATCATTCCC-3′), which includes a PflmI site of PS1 cDNA. The second PCR was performed with the primers, C99-PS1-1f and C99-PS1-2r, and a mixture of the PCR products of the first PCR-1 and first PCR-2 as templates. C99-PS1D385A, C99-PS1G266S, C99-PS1R278I and C99-PS1L435H were generated using a Quick-change site directed mutagenesis kit (Stratagene, CA, USA). C99-PS2 was generated by a PCR-based method as C99-PS1. Primers for the first PCR-1 were: C99-PS2-1f, 5′-ACCCAAGCTTCACAGCTAGCGCACTCGGTG, and C99-PS2-1r, 5′-CATTAGGGACGTCCGCTGCTCAAAGAACTTG. Primers for the first PCR-2 were: C99-PS2-2f, 5′-CAAGTTCTTTGAGCAGCGGACGTCCCTAATG, and C99-PS2-2r, 5′-GCTCTAGAGTAAAACTATACAACTGCATCC. The sequence of each mutant cDNA was verified by sequencing. Details of primer sequences and PCR conditions for all cloning work are available on request. A series of deletion constructs of C99-PS1 and C99-PS1R278I were also generated using PCR-based methods and a Quick-change site directed mutagenesis kit (Stratagene, CA, USA). F-NEXT C-PS1 (Figure S3) was generated by PCR-based method, as with C99-PS1. The primers used were: n-ps1-f, 5′-CCGGATATCGTCTGAGGACAACCACCTG, n-ps1-r, 5′-CCGCTCGAGCTAGATATAAAATTGATGGAATGC. C99-PS2 was generated by PCR-based method, as with C99-PS1.

### Cell Culture and Transfection

Transformed fibroblasts derived from mouse embryos with homozygous deletions of *PSEN1* and *PSEN2* alleles (PS dKO MEF) [19] were kindly gifted by Dr. Bart De Strooper and Dr. Alexandra Tolia, and cultured in Dulbecco's modified Eagle's medium (Nacalai Tesque, Kyoto, JAPAN) containing 10% fetal bovine serum (Biowest). Transient transfection of C99-PS1 chimeras in PS dKO MEF was performed using Fugene6 (Roche). PS dKO MEF was plated onto 6-well dishes. The next day, ∼50% confluent cells were transfected with 2 µg plasmid. Then 48 hours later, media were collected with PMSF, a protease inhibitor, and cells were lysed in lysis buffer (50 mM Tris-HCl (pH 7.4), 150 mM NaCl, 5 mM EDTA, 0.5% Nonidet P-40, 0.5% deoxycholate, 0.25% SDS and a protease inhibitor cocktail). C99-PS1 was transiently expressed in COS cells using Lipofectamine (Invitrogen). A dipeptidic γ-secretase inhibitor, N-[N-(3,5-difluorophenacetyl)-L-alanyl]-S-phenylglycine *t*-butyl ester (DAPT), was purchased from Calbiochem. Stable human embryonic kidney (HEK) 293 pools (Figure S5) were generated by transfecting cells with empty bicistronic vector (pIRE1 puro; Clontech, Mountain View, CA, USA), or pIRE1 puro containing C99-PS1wt, C99-PS1D385A, or C99-PS1G266S, and stable transfectants were selected in medium containing 2.5 µg/ml puromycin. To generate stable cell lines expressing F-NEXTΔC-PS1 (Figure S3), HEK 293 cells were transfected with cDNAs encoding F-NEXTΔC-PS1

### Antibodies

PS1 NT antibody is a monoclonal antibody that recognizes amino acid residues 21–80 (CHEMICON, CA). PS1 loop antibody is a monoclonal antibody that recognizes amino acid residues 263–378 (CHEMICON, CA). 6E10, an anti-Aβ antibody, was purchased from Sigma. For F-NEXTΔC-PS1, PS1 N-terminal antibodies, used in Figure S3, recognize the N-terminal of PS1.

### Western Blot

SDS-PAGE was carried out on Tris/Tricine gel (Invitrogen, Carlsbad, CA, USA). Samples were mixed with 2×SDS sample buffer (Invitrogen) and incubated at 37°C for 30 min immediately prior to electrophoresis. The samples were transferred onto a polyvinylidene difluoride (PVDF) membrane (Millipore, Bedford, MA, USA). Western blotting was performed using the antibodies described above. Enhanced ChemiLuminescence (ECL) was used as the detection system.

### Aβ ELISA

Analysis of secreted Aβ40 and 42(43) in the medium of PS dKO MEF expressing C99-PS1 chimeras was performed using a human-specific Aβ ELISA (Wako, Japan). According to the manufacturer's information, the sensitivity of human specific Aβ40 and 42 ELISA is 0.12 and 0.08 pM, respectively. DAPT treatment clearly decreased Aβ 40 and 42 levels from the basal levels in cells expressing the fusion proteins (Figure S4D, E). Moreover, the deletion mutants C99/PS1Δ37/Δ71 and Δ37/Δ72, which have defects in the transmembrane I domain of PS, secreted significantly less Abeta42 than did C99-PS1 (Figure S4G). These results indicate that our assay had sufficient specificity and sensitivity to detect different values of Aβ at these low levels. Because PS(−/−) cells have no γ-secretase activity and PS(−/−) cells have no human Aβ, the value of [Aβ level of vector plus Aβ level of PS1wt]/2 was set as 0.

### Statistical Analysis

All values are expressed as mean ± SEM. For comparisons of the means between two groups, the data were statistically analyzed by Student's *t*-test. Comparisons of the means among three or more groups were performed by analysis of variance (ANOVA). Values of *p* less than 0.05 were considered significant.

## Results

### Fusion of APP CTF to presenilin 1 N-terminus produces Aβ

**Figure 1:**
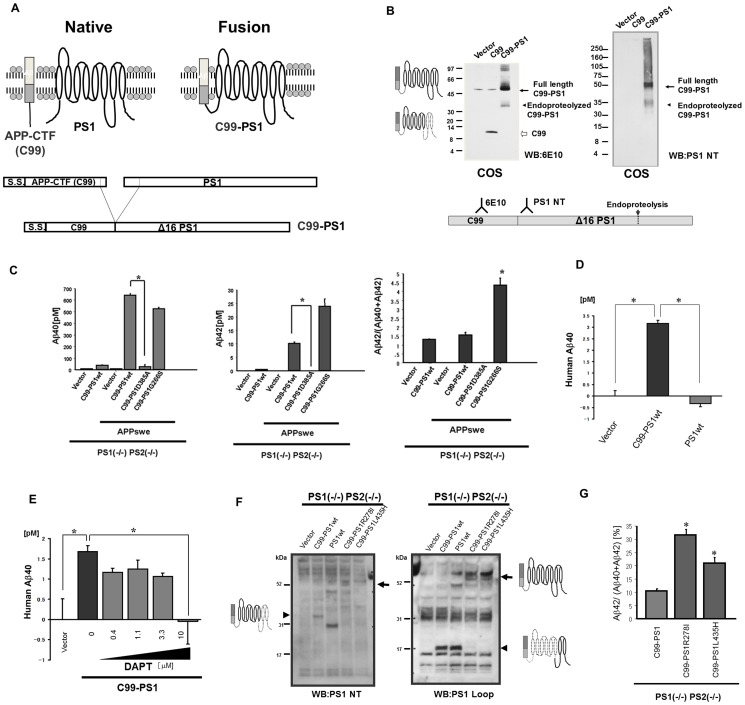
Fusion of APP CTF to PS1 N-terminus produces Aβ, while fusion of APP CTF to FAD-linked PS1 variant overproduces Aβ42. **Panel A:** Design of C99-PS1 fusion protein. The C-terminus of C99 and N-terminus of PS1 were fused with deletion of the last several amino acids of C99 and the first 16 amino acids of PS1. S.S., signal sequence. **Panel B:** Expression of C99-PS1 wild-type fusion protein in COS cells. Left panel: Western blot analysis of C99-PS1 with anti-Aβ antibody, 6E10. Arrow indicates full length C99-PS1. Arrowhead indicates endoproteolysed C99-PS1. Right panel: Western blot analysis of C99-PS1 with anti-PS1 NT antibody, which recognizes amino acid residues 21–80 of PS1. Arrow indicates full length C99-PS1. Arrowhead indicates endoproteolysed C99-PS1. **Panel C:** Aβ40 level (left), Aβ42 level (middle), and Aβ42/(Aβ40+Aβ42) ratio (right) in medium of PS1 (−/−) PS2 (−/−) transiently transfected with vector, C99-PS1, APPswe+vector, APPswe+C99-PS1wt, APPswe+C99-PS1D385A, or APPswe+C99-PS1G266S. * *p*<0.05. **Panel D:** Human Aβ40 level in medium of PS1 (−/−) PS2 (−/−) transiently transfected with vector, C99-PS1, or PS1wt analyzed by human specific Aβ40 ELISA. * *p*<0.05. **Panel E:** Human Aβ40 level in medium of PS1 (−/−) PS2 (−/−) transiently transfected with vector or C99-PS1 treated with 0, 0.4, 1.1, 3.3, and 10 µM DAPT analyzed by human specific Aβ40 ELISA. * *p*<0.05. Note that DAPT treatment decreased Aβ40 secreted from PS (−/−) cells expressing the fusion protein. **Panel F:** Western blot analysis of expression of C99-PS1, C99-PS1R278I or C99-PS1L435H in PS1 (−/−) PS2 (−/−) cells with anti-PS1 NT (left) or anti-PS1 loop (right) antibodies. In the left panel, arrowhead indicates endoproteolysed C99-PS1 NTF, while arrow indicates full length C99-PS1R278 and L435H. In the right panel, arrowhead indicates endoproteolysed C99-PS1 CTF, while arrow indicates full length C99-PS1R278 and L435H. **Panel G:** Aβ42/(Aβ40+Aβ42) ratio in medium of PS1 (−/−) PS2 (−/−) cells transiently expressing C99-PS1, C99-PS1R278I or C99-PS1L435H. * *p*<0.05.

A PCR-based method was used to generate a construct that encodes the C99-PS1 fusion protein, where the C-terminus of C99 and the N-terminus of PS1 were fused, with deletion of the last three amino acids of C99 and the first 16 amino acids of PS1 (Figure 1A). Dominant negative D385A or FAD-linked G266S mutations of PS1 were introduced into the C99-PS1 fusion protein. First, we checked the successful generation and expression of the fusion plasmid in COS cells, which are often used for this purpose. When transiently expressed in COS cells, C99-PS1 fusion protein was detected as an ∼50 kDa polypeptide using 6E10, a monoclonal antibody (mAb) raised against the human Aβ sequence. Presenilins undergo endoproteolysis within the loop region connecting transmembrane domains 6 and 7, resulting in an ∼27–30 kDa NH_2_-terminal fragment (NTF) and an ∼16–20 kDa COOH-terminal fragment (CTF) [20]. Consistent with endoproteolysis of the C99-PS1 fusion protein, we found that 6E10 mAb detected an ∼35 kDa NTF (Figure 1B). PS1NT mAb, which is raised against a peptide sequence behind the 17^th^ amino acid, also reacted with these ∼50 kDa and ∼35 kDa species, indicating that the C99-PS1 fusion protein undergoes endoproteolysis as observed in wild-type PS1 protein (Figure 1B). The stability of this fusion protein was comparable to that of PS1 protein, as suggested by the experiments using cycloheximide (30 µg/ml) (Figure S2). Moreover, we did not observe polypeptides smaller than 30 kDa in size when blots were probed with 6E10 mAb, indicating no fragmentation of APP CTF from the fusion protein. Immunoblot analysis using a mAb raised against the PS1 loop domain revealed that PS1 CTF was also detected in fibroblasts derived from PS1 (−/−) PS2 (−/−) mouse embryos (PS dKO MEF), transfected with the C99-PS1 construct (Figure 1F). Compared to COS cells, when C99-PS1 plasmid was transfected into PS dKO cells, we did not observe 6E10 immunoreactivity with a molecular weight corresponding to PS1NTF, suggesting efficient cleavage in these cells (Figure S1). These results suggest that the C99-PS1 fusion protein undergoes endoproteolysis similarly to wild-type PS1 protein.

Next, we examined whether the C99-PS1 fusion protein can restore the loss of γ-secretase function in PS dKO MEF. We co-transfected PS dKO MEF with plasmids encoding the APP “Swedish” mutant (APPswe) along with an empty vector, C99-PS1, C99-PS1D385A or C99-PS1G266S plasmid. Co-transfection of C99-PS1 and APPswe produced Aβ40 and Aβ42 (Figure 1C), while PS1D385A, a dominant negative mutation, failed to do so. Co-transfection of C99-PS1G266S, which harbors an FAD-linked PS1 mutation, and APPswe overproduced Aβ42 (Figure 1C). Taken together, these results indicate that the C99-PS1 fusion protein restores γ-secretase activity in PS dKO MEF. Next, we investigated whether C99 within C99-PS1 could be a substrate for γ-secretase. Analysis using a human Aβ40-specific ELISA clearly showed that expression of C99-PS1, but not PS1, produced human Aβ40 (Figure 1D), suggesting that C99 within C99-PS1 undergoes *in cis* processing by γ-secretase-like activity. To confirm whether a substrate fused to PS1 could be cleaved, we also performed an experiment to investigate whether the fusion protein of F-NEXTΔC, a mouse Notch1 [15], another type I membrane protein, derivative that lacks the majority of its extracellular and intracellular domains [21], to PS1 produces an Aβ-like peptide, Nβ [22]. We found that Nβ is indeed produced in K293 cells stably expressing F-NEXTΔC-PS1, suggesting that a substrate fused to PS1 could be cleaved (Figure S3). To confirm whether Aβ was indeed produced by γ-secretase processing in PS dKO MEF, we treated the cells with the γ-secretase inhibitor, DAPT. We found that DAPT inhibited Aβ production in a dose-dependent manner (Figure 1E). Taking these results together, the PS1 protein module within the C99-PS1 fusion protein functions to restore γ-secretase activity in PS dKO MEF, and the C99 sequence serves as a substrate for γ-secretase processing.

### Fusion of APP CTF to FAD-linked presenilin variant N-terminus overproduces Aβ42(43)

To test whether C99-PS1 harboring FAD-linked PS1 mutations can overproduce Aβ42(43), we introduced PS1R278I, in addition to L435H, which was found to effectively overproduce Aβ42(43), using a random mutagenesis screen of PS1 [23], [24]. Western blotting analysis detected full-length C99-PS1R278I and C99-PS1L435H proteins at ∼55 kDa in SDS-PAGE, because PS1R278I and L435H are reported not to be endoproteolysed within the PS1 loop region (Figure 1F). Analysis by Aβ ELISA showed that both C99-PS1R278I and C99-PS1L435H overproduced Aβ42(43) (Figure 1G). Taking these results together, C99-PS1R278I and C99PS1L435H, which were not endoproteolyzed within the PS1 loop region, overproduced Aβ42(43). Thus, we consider that this chimeric protein mimics the native interaction between APP CTF and presenilin.

### Differential effects of deletion in C99-PS1 wild type on Aβ40 and 42 production

**Figure 2:**
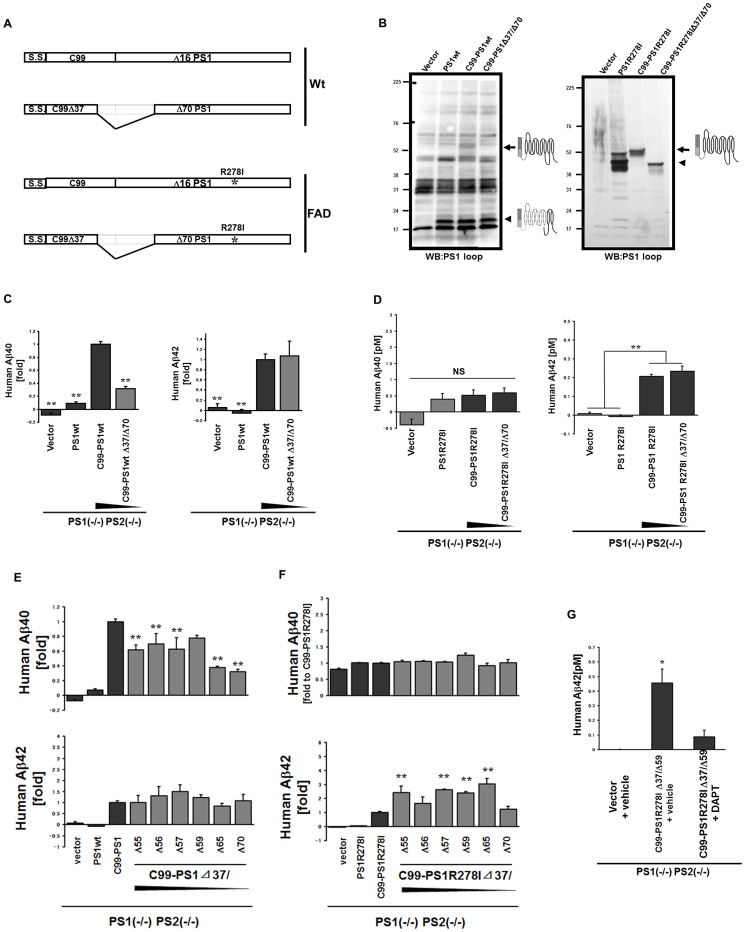
Differential effects of deletion in C99-PS1 wild type on Aβ40 and 42 production. **Panel A**: Design of deletion mutants of C99-PS1 and C99-PS1R278I. **Panel B**: Western blot analysis of PS1 (−/−) PS2 (−/−) cells transfected with deletion mutants of C99-PS1 and C99-PS1R278I with anti-PS1 loop antibody. In the left panel, arrowhead indicates endoproteolysed C99-PS1 CTF, while arrow indicates full length C99-PS1. In the right panel, arrow indicates full length C99-PS1R278. Arrow indicates C99-PS1R278I deletion product. **Panel C, D**: Analysis of Aβ40 level (left) and Aβ42 level (right) by Aβ ELISA in medium of PS1 (−/−) PS2 (−/−) cells transfected with C99-PS1 deletion mutants (C) or C99-PS1R278I (D). ** *p*<0.01. **Panel E**: Analysis of Aβ40 level (upper panel) and Aβ42 level (lower panel) by Aβ ELISA in medium of PS1 (−/−) PS2 (−/−) cells transfected with serial deletion mutants of C99-PS1.The value of [Aβ level of C99-PS1]-[Aβ level of vector plus Aβ level of PS1wt]/2 was set as 1. ** *p*<0.01. **Panel F**: Analysis of Aβ40 level (upper panel) and of Aβ42 level (lower panel) by Aβ ELISA in medium of PS1 (−/−) PS2 (−/−) cells transfected with serial deletion mutants of C99-PS1R278I. The value of the Aβ level of C99-PS1R278I was set as 1 for Aβ40 (upper panel). Note that C99-PS1R278I did not produce Aβ40. The value of [Aβ level of C99-PS1R278I]-[Aβ level of vector plus Aβ level of PS1wt]/2 was set as 1 for Aβ42 (lower panel). ** *p*<0.01. **Panel G**: Analysis of Aβ42 level by Aβ ELISA in medium of PS1 (−/−) PS2 (−/−) cells transfected with C99 Δ37-PS1R278I Δ59 treated with or without γ-inhibitor, DAPT. * *p*<0.05. Note that DAPT treatment decreased Aβ42 secreted from PS (−/−) cells expressing the fusion protein.

To change the molecular interaction between APP and PS1, we made sequential deletions of the hydrophilic region between the transmembrane domain of C99 and transmembrane I domain of PS1 in the chimeric proteins. We characterized Aβ40/42 production in PS dKO MEF transiently expressing deletion mutants of C99-PS1 WT and R278I (Figure 2A, B). Aβ40 production was reduced when C99Δ37-PS1Δ70 was transiently expressed in PS dKO MEF as compared with expression of C99-PS1 (Figure 2C), whereas Aβ42 production was unaffected. Moreover, Aβ42 production did not significantly decrease when C99Δ37-PS1R278IΔ70 was transiently expressed in PS dKO MEF compared to C99-PS1R278I (Figure 2D). These results indicate that the APP/PS interaction is differentially regulated during Aβ40 and 42 production. Next, to confirm this hypothesis, we made a series of shorter deletions within the PS1 N-terminus and chose clones with Aβ42 levels equivalent to that with the original C99-PS1 fusion protein, to exclude clones with low expression of the transgene from further analysis. We found differential effects of serial deletions in C99-PS1 on Aβ40 and 42 production. Deletion of C99-PS1 decreased Aβ40, while it did not decrease Aβ42 (Figure 2E). Moreover, deletion of C99-PS1R278I also did not decrease Aβ42 (Figure 2F). While C99-PS1R278I does not produce Aβ40 as shown in Fig. 2D, deletions within C99-PS1R278I did not affect Aβ40 production (Figure 2F). These data support the hypothesis that the APP/PS interaction is differentially regulated during Aβ40 and 42 production.

**Figure 3:**
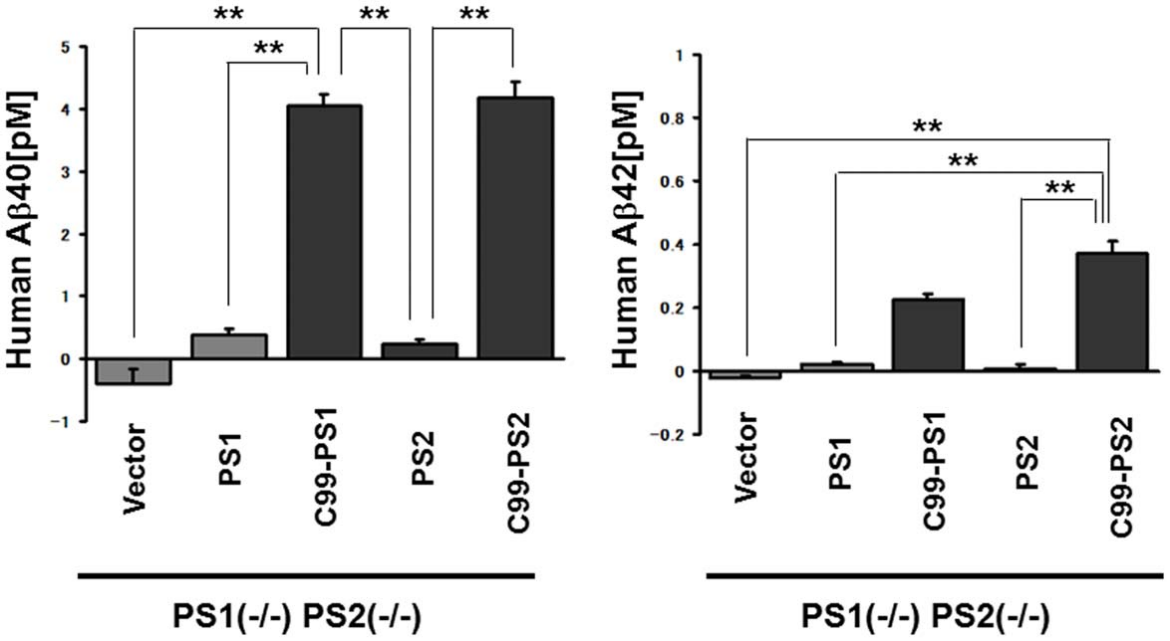
Fusion of APP CTF to PS2 N-terminus produces Aβ. Analysis of Aβ40 level (left) and Aβ42 level (right) by Aβ ELISA in medium of PS1 (−/−) PS2 (−/−) cells transfected with vector, PS1, C99-PS1, PS2, or C99-PS2. ** p<0.01.

Next, to confirm that Aβ42 production by the C99-PS1R278I fusion protein was strictly γ-secretase dependent, and not due to non-specific degradation, we treated dKO MEF expressing C99-PS1R278IΔ38/Δ59 with a selective γ-secretase inhibitor, DAPT. Aβ42 production was inhibited by DAPT in dKO MEF expressing C99-PS1R278IΔ38/Δ59 (Figure 2G), suggesting that Aβ42 production by the C99-PS1R278I fusion protein was indeed γ-secretase-dependent. Finally, we generated C99-PS2, a fusion protein of C99 and the PS1 homolog that can also function as the catalytic subunit of γ-secretase, and found that this chimeric protein also produced Aβ, when expressed in dKO MEF (Figure 3).

## Discussion

**Figure 4:**
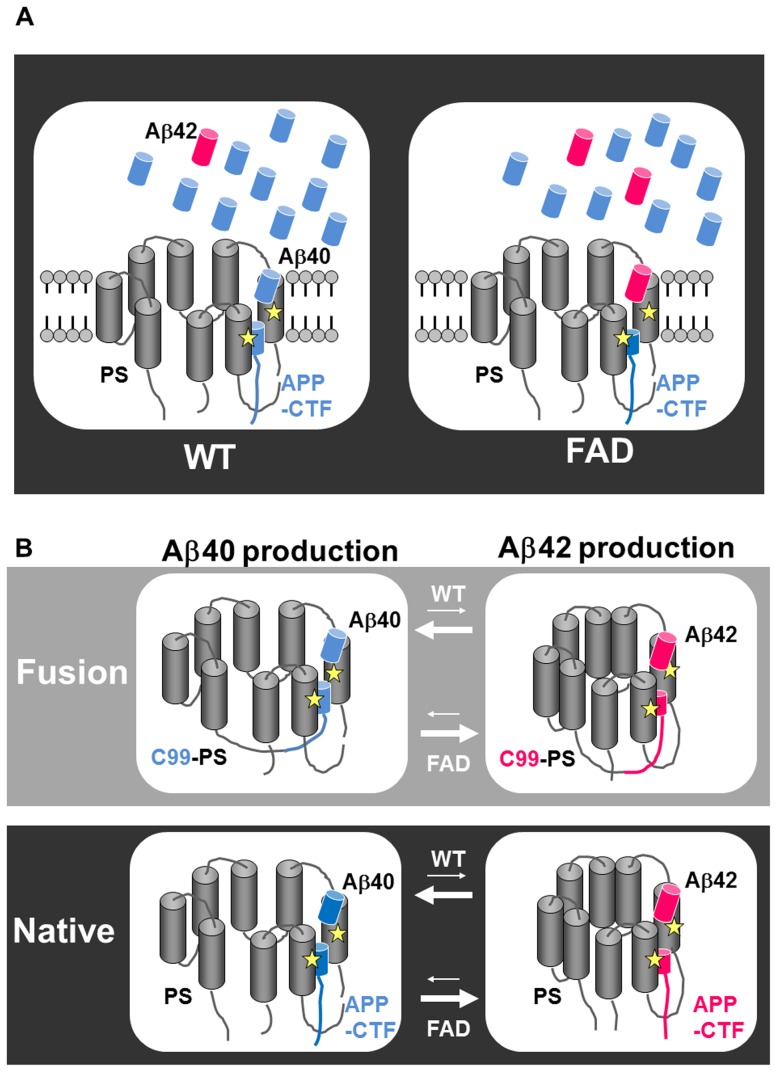
Model depicting differential regulation of APP-CTF/PS1 interaction during Aβ40 and Aβ42 production. Aβ40/Aβ42 are produced in a ratio of ten to one by wild type PS, and Aβ42 is overproduced by FAD-linked PS variants (A). Hypothetical model of differential regulation of APP-CTF/PS1 interaction during Aβ40 and Aβ42 production (B), suggested by our data. WT, wild type; FAD, familial Alzheimer disease.

The mechanisms by which Aβ40 and Aβ42 peptides are produced in a ratio of ten to one by wild type PS and Aβ42 is overproduced by FAD-linked PS variants are not clearly elucidated (Figure 4A). To understand the mechanisms by which Aβ40 and Aβ42 are produced, we generated chimeras by fusing APP C99 with the N-terminus of presenilin (PS1 or PS2). We found that expression of this fusion protein in MEF lacking PS1 and PS2 expression generated Aβ. Furthermore, a fusion protein harboring the FAD-linked PS1 mutation overproduced Aβ42. To experimentally alter the intermolecular interactions between the C99 and PS1 modules within the fusion protein, we introduced sequential deletions between C99 and PS1. We found differential effects of deletion in C99-PS1 wild type on Aβ40 and 42 production. While deletions of C99-PS1 decreased the level of Aβ40, there was no effect on the level of Aβ42. Moreover, deletions within C99-PS1R278I also did not decrease Aβ42. These data suggest differential regulation of the APP/PS interaction during Aβ40 and 42 production.

Based on our findings, we suggest that the chimeric proteins characterized in this study mimic the native interaction between APP CTF and presenilin, and would be useful tools for further exploration. First, we showed that fusion of APP CTF to the N-terminus of PS1 produced Aβ by intrinsic processing upon reconstituion of γ-secretase activity in PS dKO cells. Moreover, a γ-secretase inhibitor, DAPT, inhibited Aβ production, suggesting that fusion of APP CTF to the N-terminus of PS1 produced Aβ in a γ-secretase-dependent manner. We also found that Nβ is indeed produced in K293 cells stably expressing F-NEXTΔC-PS1, the fusion protein of F-NEXTΔC, a mouse Notch1 derivative that lacks the majority of its extracellular and intracellular domains [21], to PS1 suggesting that a substrate fused to PS1 could be cleaved (Figure S3). The maximum amount of Aβ that could be produced would be no greater than the amount of APP-PS1 fusion protein produced. The expression level of PS1 is regulated by a limiting factor, i.e. the γ-secretase complex. Thus, this might be the reason why the amount of Aβ that is produced in cells expressing only the fusion protein is very low. However, DAPT treatment clearly decreased Aβ40 and 42 levels from the basal levels in cells expressing the fusion proteins (Figure S4D, E). Moreover, the deletion mutants C99/PS1Δ37/Δ71 and Δ37/Δ72, which have defects in the transmembrane I domain of PS, secreted significantly less Abeta42 than did C99-PS1 (Figure S4G). These results indicate that our assay had sufficient specificity and sensitivity to detect different values of Aβ even at these low levels. Second, fusion of APP CTF to the FAD-linked PS1 variant overproduced Aβ42 (Figure 1). Since APP CTF and PS are transmembrane proteins, fusion of the APP cytosolic C-terminus to the hydrophilic N-terminus of presenilin might allow them to maintain the right positional relationship on the membrane bilayer via hydrophobic transmembrane domains. This strategy could be utilized to investigate the mechanism of other intra-membranous cleavage or other associations between two proteins within or on the membrane. Recent findings that the first transmembrane domain of PS1 plays an important role in the enzyme/substrate relationship [25], [26] might support our strategy of fusion of APP CTF adjacent to the first transmembrane domain of presenilin. Third, we found differential effects of deletion in C99-PS1 wild type on Aβ40 and 42 production. Deletion of residues between the transmembrane domain of APP and the first transmembrane domain of PS1 decreased Aβ40 production, while having no effect on Aβ42 production. Moreover, deletions within C99-PS1R278I also did not reduce Aβ42(43) production. It is reported that FAD-linked PS1 mutations cause alterations in the conformation of PS and interactions with APP [27]. Interestingly, heterogeneity of the γ-secretase complex, i.e. Aph1B or Aph1A, causes a change in the relative ratio of Aβ40 and 42 production [28], associated with conformational change of PS1. It is essential to investigate whether cleavage of the C99-PS1 fusion protein requires binding to the other members of the γ-secretase complex. We observed replacement of endogenous PS1 when the fusion proteins C99-PS1 and F-NEXTΔC-PS1 were stably expressed in stable K293 cells (Figures S3, S5). These data suggest that the fusion protein is incorporated into the γ-secretase complex. We are currently investigating whether the production of Aβ from the C99-PS1 fusion protein requires binding to the other members of the γ-secretase complex. In addition, NSAIDs reduce Aβ42 production, also associated with alterations in the conformation of PS and interactions with APP. Our data suggest that conformational change of the APP/PS interaction might underlie cleavage site selection leading to Aβ40 and 42 production (Figure 4B). It might be also interesting to test the effects of the FAD-linked mutations in the APP gene in this fusion system. Introduction of APP Iberian [29] mutation which overproduce Aβ42 [30] might result in a similar effect of deletion of residues between C99 and PS1 on Aβ production to C99-PS1R278I. It is proposed that APP CTF is cleaved first at the membrane-cytoplasm boundary, producing two longer Aβ species, Aβ48 and Aβ49, which are processed further by releasing three residues at each step to produce Aβ42 and Aβ40, respectively [31], suggesting that the mechanisms by which Aβ42 and Aβ40 are generated utilize differential substrate-catalytic site interaction to yield two distinct product lines. Our data presented here are also not inconsistent with this hypothesis. Taken together, these data suggest that the APP/PS interaction is differentially regulated during Aβ40 and 42 production.

In summary, we generated fusion proteins of APP CTF to the N-terminus of PS and observed differential regulation of the APP/PS interaction during Aβ40 and 42 production. Because the crystal structures of other intramembrane proteases have been reported [32], [33], further analysis including high resolution of crystal structures or NMR of C99-PS1 chimeras would provide further insights into the mechanisms by which Aβ40/Aβ42 are produced in a ratio of ten to one by wild type PS and Aβ42 is overproduced by FAD-linked PS variants, and open the door to structure-based design of pharmacological modulators of this protease.

## Supporting Information

Text S1 (DOCX)

Figure S1 **Comparison of expression level of C99-PS1 in COS cells and PS(−/−) cells.** A higher level of protein expression was observed in COS cells than in PS(−/−) cells. Note that no reactivity with 6E10 antibody was observed in cells that expressed C99-PS1 fusion protein in PS(−/−) cells, with even high exposure.
(TIF)

Figure S2 **Stability of C99-PS1 fusion protein in COS cells.** The stability of the fusion protein was investigated using cycloheximide (30 µg/ml). Note that that stability of C99-PS1 was comparable to that of PS1. We observed that the immunoreactivity of the fusion protein was not very different between PS1 NT and 6E10 in COS cells. However, this result does not exclude that the fusion protein is being cleaved efficiently, because it is highly overexpressed in COS cells.
(TIF)

Figure S3 **Generation of F-NEXTΔC-PS1 and detection of Nβ.** The fusion protein of Notch1, another type I membrane protein, to PS1 was generated. F-NEXTΔC-PS1/K293-clone #15 expressed a high level of full-length F-NEXT-ΔC-PS1, and replacement of endogenous PS1 NTF and PS2 by full-length F-NEXT-ΔC-PS1 was observed in this cell line, but less replacement was observed in clone #4 with low expression of full-length F-NEXT-ΔC-PS1 (**left panel**). IP-Mass experiment revealed that Nβ was secreted in F-NEXTΔC-PS1/K293-clone #15. Thus, we confirmed that the fusion protein of flag-tagged NotchΔE fused to PS1 could be cleaved, resulting in Nβ secretion (**right panel**), suggesting that substrate fused to PS1 could be cleaved.
(TIF)

Figure S4 **Validation of Aβ ELISA.** **Panel A:** Standard curve for Aβ40 obtained using synthetic Aβ40 peptides. **Panel B:** Standard curve for Aβ42 obtained using synthetic Aβ42 peptides. **Panel C:** Comparison of secreted Aβ from PS dKO cells expressing C99-PS1 using human/rat Aβ ELISA (**left panel**) and human-specific Aβ ELISA (**right panel**). * *p*<0.05. **Panel D:** Human Aβ40 level in medium of PS dKO cells transiently transfected with vector or C99-PS1 treated with 0, 0.4, 1.1, 3.3, and 10 µM DAPT analyzed by human-specific Aβ40 ELISA. * *p*<0.05. Note that DAPT treatment decreased Aβ40 secreted from PS dKO cells expressing the fusion protein. **Panel E:** Analysis of Aβ42 level by Aβ ELISA in the medium of PS dKO cells transfected with C99 -PS1R278I Δ37/Δ59 treated with or without γ-inhibitor, DAPT. * *p*<0.05. Note that DAPT treatment decreased Aβ42 secreted from PS (−/−) cells expressing the fusion protein. **Panel F:** Analysis of Aβ level by Aβ ELISA in the medium of PS dKO cells transfected with C99/PS1Δ37/Δ65. Note that the deletion mutants of C99/PS1Δ37/Δ65 showed decreased production of Aβ42, but not Aβ40 compared to C99-PS1. * *p*<0.05, ** *p*<0.01. **Panel G:** Analysis of Aβ level by Aβ ELISA in the medium of PS dKO cells transfected with C99/PS1Δ37/Δ71 and Δ37/Δ72. Note that the deletion mutants of C99/PS1Δ37/Δ71 and Δ37/Δ72, which have defects in the transmembrane I domain of PS, showed disrupted production of both Aβ40 and 42 compared to C99-PS1. * *p*<0.05, ** *p*<0.01.
(TIF)

Figure S5 **Replacement of endogenous PS1 by fusion protein.** Stable human 293 pools were generated by transfecting cells with vector, or C99-PS1wt, C99-PS1D385A, or C99-PS1G266S. Note that endogenous PS1 CTF was replaced by C99-PS1D385A, which is the full-length dominant negative form.
(TIF)
