# Correction: Differential Regulation of Amyloid Precursor Protein/Presenilin 1 Interaction during Ab40/42 Production Detected Using Fusion Constructs

**DOI:** 10.1371/annotation/5cac3524-f2a4-4997-ba21-a5f1d8551f59

**Published:** 2012-11-14

**Authors:** Naoyuki Sato, Masayasu Okochi, Mitsuru Shinohara, Gopal Thinakaran, Shuko Takeda, Akio Fukumori, Motoko Shinohara-Noma, Mari Mori-Ueda, Hizuki Hamada, Masatoshi Takeda, Hiromi Rakugi, Ryuichi Morishita

The term "Aβ40/42" is incorrect in the article title.

The correct title is: Differential Regulation of Amyloid Precursor Protein/Presenilin 1 Interaction during Aβ40/42 Production Detected Using Fusion Constructs

The correct citation is: Sato N, Okochi M, Shinohara M, Thinakaran G, Takeda S, et al. (2012) Differential Regulation of Amyloid Precursor Protein/Presenilin 1 Interaction during Aβ40/42 Production Detected Using Fusion Constructs. PLoS ONE 7(11): e48551. doi:10.1371/journal.pone.0048551 

