# Supplementary material for: Differential Regulation of Amyloid Precursor Protein/Presenilin 1 Interaction during Ab40/42 Production Detected Using Fusion Constructs
Source: PLoS One. 2012 Nov 12;7(11):e48551. doi: 10.1371/journal.pone.0048551 (PMC3495957; doi:10.1371/journal.pone.0048551)
Supplement: Text S1 — (DOCX) [file pone.0048551.s001.docx]

**Supporting Information**

**Stability of C99-PS1 fusion protein**

We performed experiments using cycloheximide (30 μg/ml) to investigate the stability of the fusion protein. We found that the stability of C99-PS1 was comparable to that of PS1 (Figure S2). We observed that the immunoreactivity of the fusion protein was not very different between PS1 NT and 6E10 in COS cells. However, this result does not exclude that the fusion protein is being cleaved efficiently, because it is highly overexpressed in COS cells. Indeed, we did not observe 6E10 immunoreactivity when the fusion protein was expressed in PS (-/-) cells (Figure S1).

**Generation of F-NEXTΔC-PS1 and detection of Nβ**

To confirm that a substrate fused to PS1 could be cleaved, we investigated whether the fusion protein of F-NEXTΔC, a mouse Notch1[[1](#_ENREF_1)], another type I membrane protein, derivative that lacks the majority of its extracellular and intracellular domains [[2](#_ENREF_2)], to PS1 produces Nβ[[3](#_ENREF_3)]. We established HEK293 cell lines stably expressing F-NEXTΔC-PS1. F-NEXTΔC-PS1/K293-clone #15 expressed a high level of full-length F-NEXT-ΔC-PS1, and replacement of endogenous PS1 NTF and PS2 by full-length F-NEXT-ΔC-PS1 was observed in this cell line, but less replacement was observed in clone #4 with low expression of full-length F-NEXT-ΔC-PS1 (Figure S3, left panel). IP-Mass experiment revealed that Nβ was secreted in F-NEXTΔC-PS1/K293-clone #15. Thus, we confirmed that the fusion protein of flag-tagged NotchΔE fused to PS1 could be cleaved, resulting in Nβ secretion (Figure S3, right panel), suggesting that substrate fused to PS1 could be cleaved.

**Methods**

***Generation of F-NEXTΔC-PS1***

F-NEXT C-PS1 was generated by PCR-based method, as with C99-PS1. The primers used were: n-ps1-f, 5’-CCGGATATCGTCTGAGGACAACCACCTG, n-ps1-r, 5’-CCGCTCGAGCTAGATATAAAATTGATGGAATGC. C99-PS2 was generated by PCR-based method, as with C99-PS1.

#### *Cell Culture and Transfection*

Human embryonic kidney (HEK) 293 cells were cultured in Dulbecco’s modified Eagle’s medium (Nacalai tesque, Kyoto, Japan) containing 10% fetal bovine serum (Biowest). Stable human 293 pools were generated by transfecting cells with empty bicistronic vector (pIRE1 puro; Clontech, Mountain View, CA, USA), or pIRE1 puro containing C99-PS1wt, C99-PS1D385A, or C99-PS1G266S, and stable transfectants were selected in medium containing 2.5 μg/ml puromycin.

To generate stable cell lines expressing F-NEXTΔC-PS1, human embryonic kidney 293 cells were transfected with cDNAs encoding F-NEXTΔC-PS1.

#### *Antibodies*

PS1 NT antibody is a monoclonal antibody that recognizes amino acid residues 21-80 (CHEMICON, CA). For F-NEXTΔC-PS1, PS1 N-terminal antibodies, used in Figure S3, recognize the N-terminal of PS1.

**References**

**1. De Strooper B, Annaert W, Cupers P, Saftig P, Craessaerts K, et al. (1999) A presenilin-1-dependent gamma-secretase-like protease mediates release of Notch intracellular domain. Nature 398: 518-522.**

**2. Tagami S, Okochi M, Yanagida K, Ikuta A, Fukumori A, et al. (2008) Regulation of Notch signaling by dynamic changes in the precision of S3 cleavage of Notch-1. Mol Cell Biol 28: 165-176.**

**3. Okochi M, Steiner H, Fukumori A, Tanii H, Tomita T, et al. (2002) Presenilins mediate a dual intramembranous gamma-secretase cleavage of Notch-1. Embo J 21: 5408-5416.**
