# Supplementary figures and images for: Differential Regulation of Amyloid Precursor Protein/Presenilin 1 Interaction during Ab40/42 Production Detected Using Fusion Constructs
Source: PLoS One. 2012 Nov 12;7(11):e48551. doi: 10.1371/journal.pone.0048551 (PMC3495957; doi:10.1371/journal.pone.0048551)

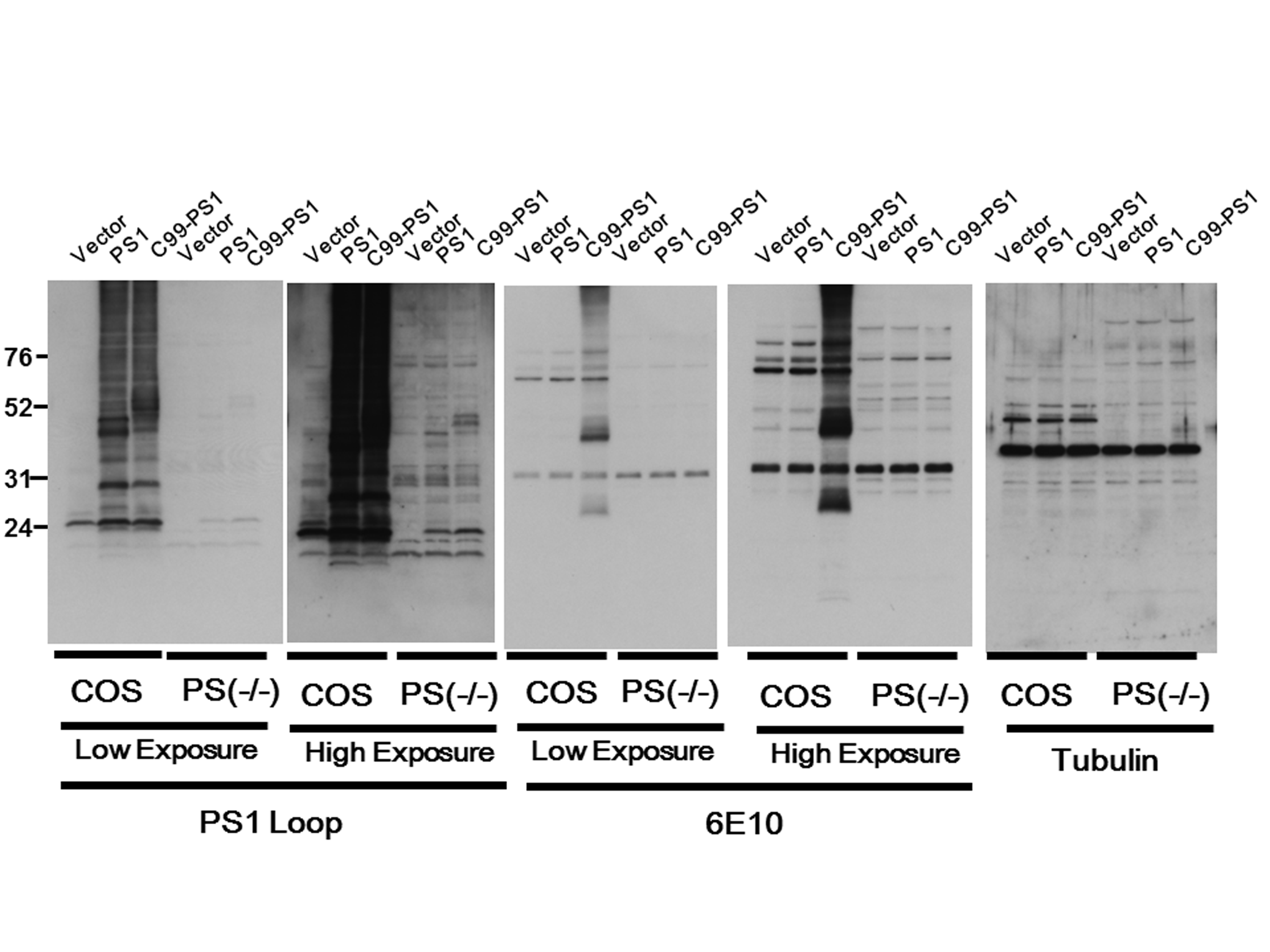

Supplement: Figure S1 — Comparison of expression level of C99-PS1 in COS cells and PS(−/−) cells. A higher level of protein expression was observed in COS cells than in PS(−/−) cells. Note that no reactivity with 6E10 antibody was observed in cells that expressed C99-PS1 fusion protein in PS(−/−) cells, with even high exposure. (TIF) [file pone.0048551.s002.tif]

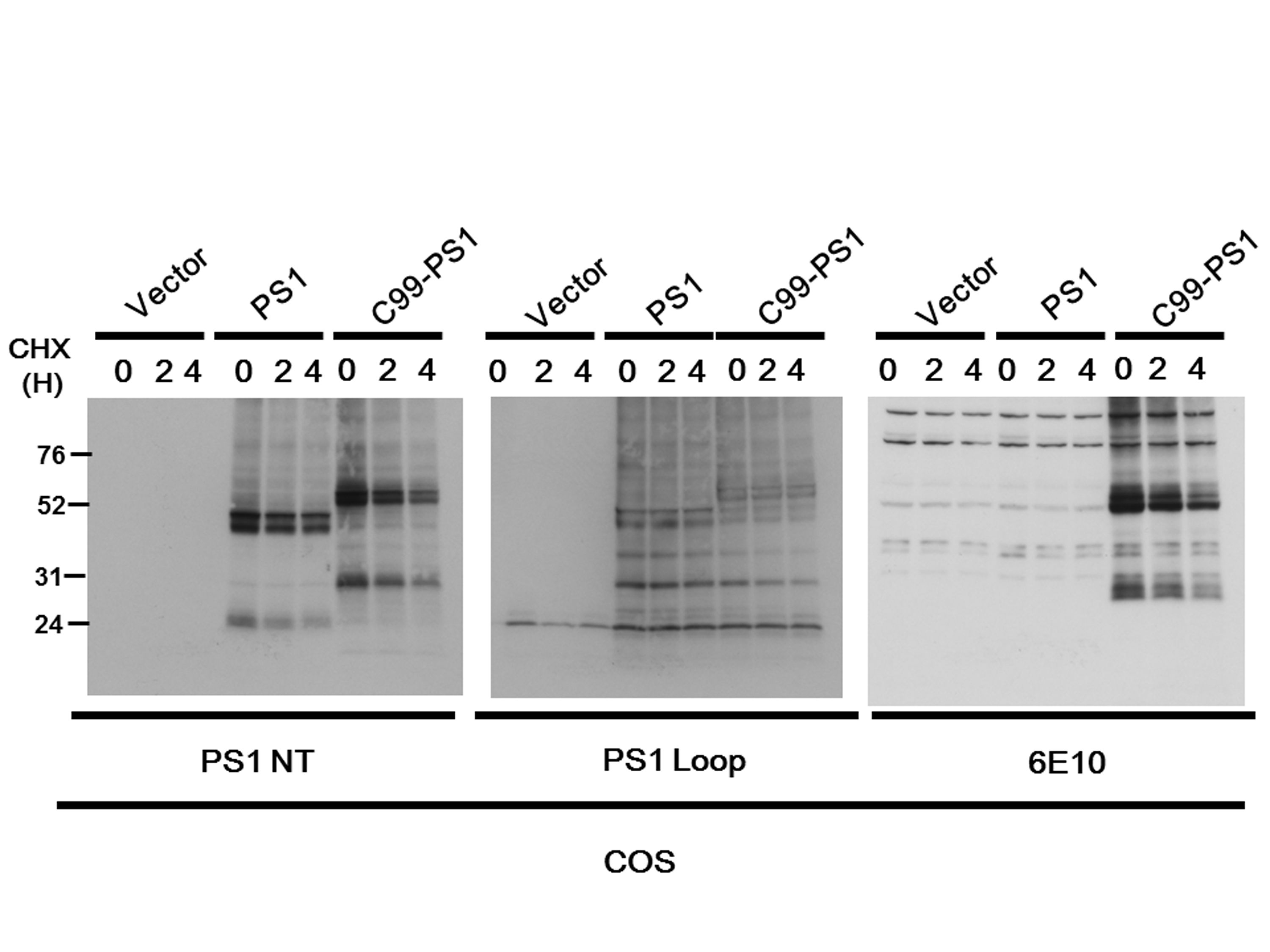

Supplement: Figure S2 — Stability of C99-PS1 fusion protein in COS cells. The stability of the fusion protein was investigated using cycloheximide (30 µg/ml). Note that that stability of C99-PS1 was comparable to that of PS1. We observed that the immunoreactivity of the fusion protein was not very different between PS1 NT and 6E10 in COS cells. However, this result does not exclude that the fusion protein is being cleaved efficiently, because it is highly overexpressed in COS cells. (TIF) [file pone.0048551.s003.tif]

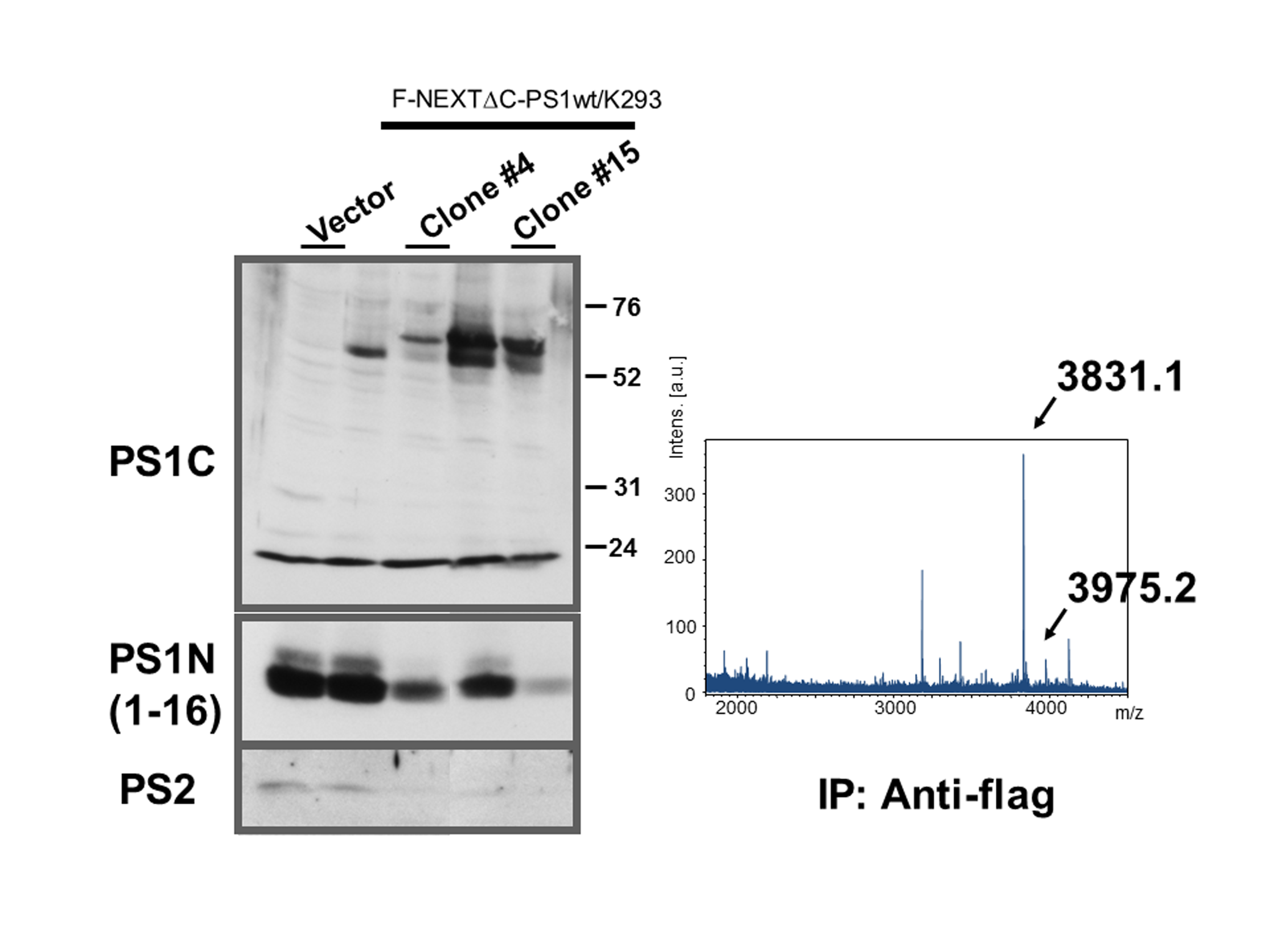

Supplement: Figure S3 — Generation of F-NEXTΔC-PS1 and detection of Nβ. The fusion protein of Notch1, another type I membrane protein, to PS1 was generated. F-NEXTΔC-PS1/K293-clone #15 expressed a high level of full-length F-NEXT-ΔC-PS1, and replacement of endogenous PS1 NTF and PS2 by full-length F-NEXT-ΔC-PS1 was observed in this cell line, but less replacement was observed in clone #4 with low expression of full-length F-NEXT-ΔC-PS1 (left panel). IP-Mass experiment revealed that Nβ was secreted in F-NEXTΔC-PS1/K293-clone #15. Thus, we confirmed that the fusion protein of flag-tagged NotchΔE fused to PS1 could be cleaved, resulting in Nβ secretion (right panel), suggesting that substrate fused to PS1 could be cleaved. (TIF) [file pone.0048551.s004.tif]

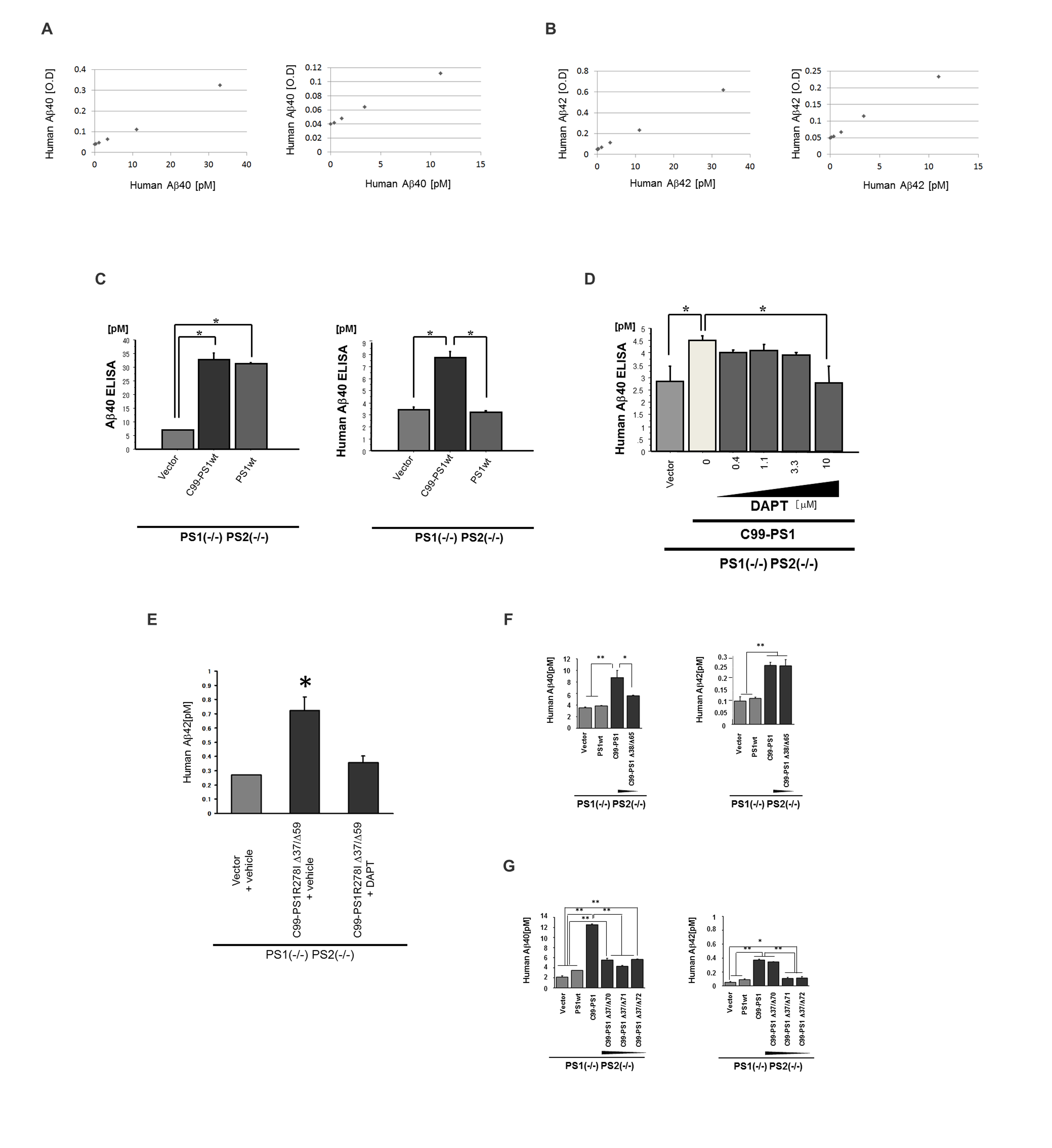

Supplement: Figure S4 — Validation of Aβ ELISA. Panel A: Standard curve for Aβ40 obtained using synthetic Aβ40 peptides. Panel B: Standard curve for Aβ42 obtained using synthetic Aβ42 peptides. Panel C: Comparison of secreted Aβ from PS dKO cells expressing C99-PS1 using human/rat Aβ ELISA (left panel) and human-specific Aβ ELISA (right panel). * p<0.05. Panel D: Human Aβ40 level in medium of PS dKO cells transiently transfected with vector or C99-PS1 treated with 0, 0.4, 1.1, 3.3, and 10 µM DAPT analyzed by human-specific Aβ40 ELISA. * p<0.05. Note that DAPT treatment decreased Aβ40 secreted from PS dKO cells expressing the fusion protein. Panel E: Analysis of Aβ42 level by Aβ ELISA in the medium of PS dKO cells transfected with C99 -PS1R278I Δ37/Δ59 treated with or without γ-inhibitor, DAPT. * p<0.05. Note that DAPT treatment decreased Aβ42 secreted from PS (−/−) cells expressing the fusion protein. Panel F: Analysis of Aβ level by Aβ ELISA in the medium of PS dKO cells transfected with C99/PS1Δ37/Δ65. Note that the deletion mutants of C99/PS1Δ37/Δ65 showed decreased production of Aβ42, but not Aβ40 compared to C99-PS1. * p<0.05, ** p<0.01. Panel G: Analysis of Aβ level by Aβ ELISA in the medium of PS dKO cells transfected with C99/PS1Δ37/Δ71 and Δ37/Δ72. Note that the deletion mutants of C99/PS1Δ37/Δ71 and Δ37/Δ72, which have defects in the transmembrane I domain of PS, showed disrupted production of both Aβ40 and 42 compared to C99-PS1. * p<0.05, ** p<0.01. (TIF) [file pone.0048551.s005.tif]

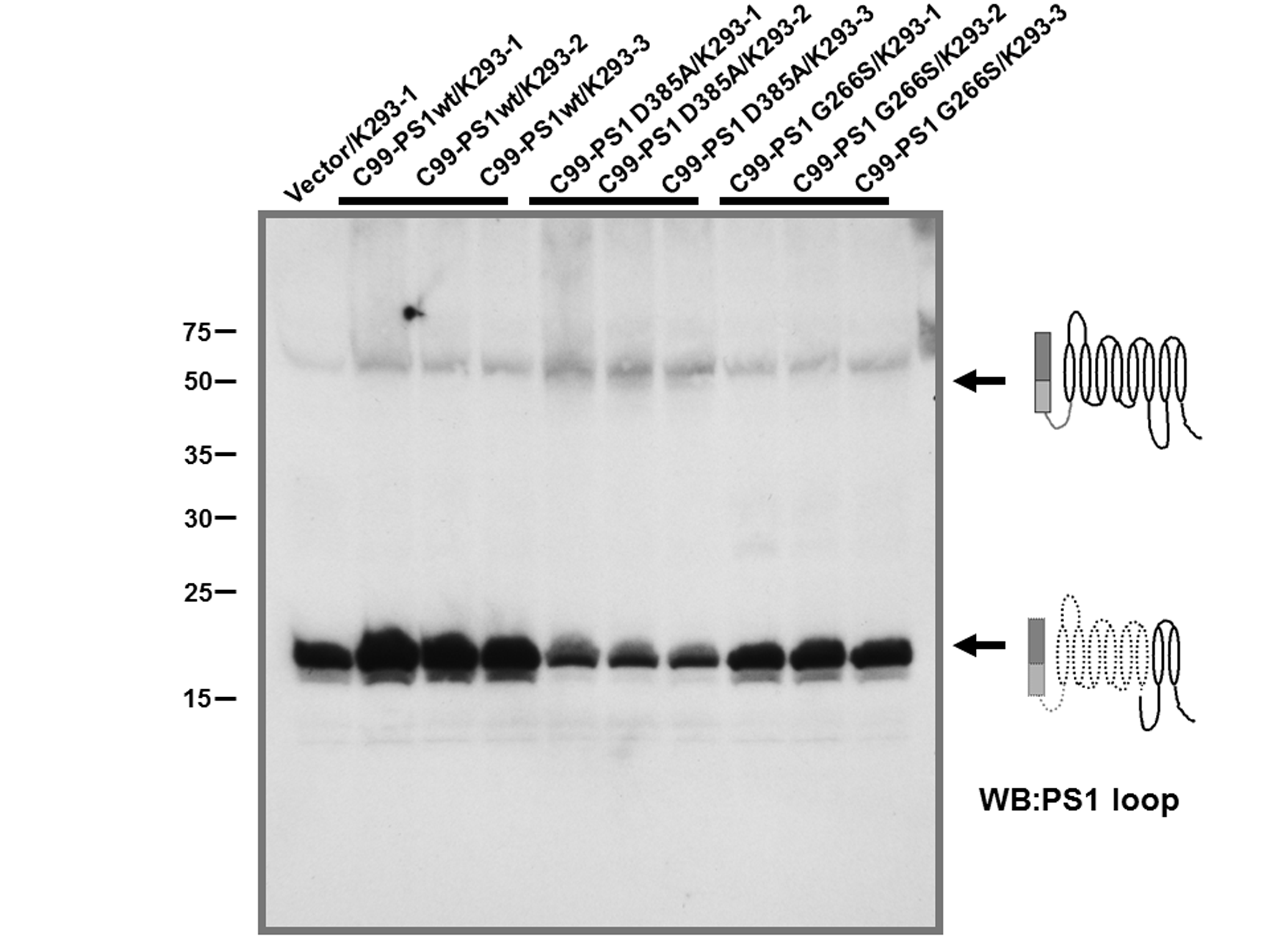

Supplement: Figure S5 — Replacement of endogenous PS1 by fusion protein. Stable human 293 pools were generated by transfecting cells with vector, or C99-PS1wt, C99-PS1D385A, or C99-PS1G266S. Note that endogenous PS1 CTF was replaced by C99-PS1D385A, which is the full-length dominant negative form. (TIF) [file pone.0048551.s006.tif]
